# Design of mirtazapine solid dispersion with different carriers’ systems: optimization, in vitro evaluation, and bioavailability assessment

**DOI:** 10.1007/s13346-023-01316-9

**Published:** 2023-03-20

**Authors:** Reem Abd Elhameed Aldeeb, Mahmoud Abd El-Ghani Mahdy, Hanan Mohamed El-Nahas, Abeer Abdelaziz Musallam

**Affiliations:** 1 Department of Pharmaceutics, College of Pharmaceutical Sciences and Drug Manufacturing, Misr University for Science and Technology, 6th of October City, Giza, 12582 Egypt; 2 Department of Pharmaceutics, Faculty of Pharmacy, Zagazig University, Zagazig, Egypt

**Keywords:** Mirtazapine, Solid dispersion, Polymers, D-optimal design, Loading efficiency, Aqueous solubility, Dissolution rate, Oral bioavailability

## Abstract

The solid dispersion technique is the most effective and widely used approach for increasing the solubility and release of drugs that have low water solubility. Mirtazapine (MRT) is an atypical antidepressant used to treat severe depression. MRT has a low oral bioavailability (about 50%) due to its low water solubility (BCS class II). The study’s goal was to determine optimum conditions for incorporating MRT into various polymer types utilizing the solid dispersion (SD) technique, with the goal of selecting the most suitable formula with the optimal aqueous solubility, loading efficiency, and dissolution rate. The D-optimal design was used to pick the optimal response. The optimum formula was subjected to physicochemical evaluation by Fourier transform infrared spectroscopy (FT-IR), differential scanning calorimetry (DSC), X-ray powder diffraction (XRPD), and scanning electron microscopy (SEM). In vivo bioavailability study was conducted on white rabbits’ plasma samples. MRT-SDs were prepared by the solvent evaporation method using Eudragit (RL-100, RS-100, E-100, L-100–55), PVP K-30, and PEG 4000 with different drug/polymer percentages (33.33%, 49.99%, and 66.66%). Results showed that the optimum formula obtained using PVP K-30 at a drug percentage of 33.33% gave a loading efficiency of 100.93%, an aqueous solubility of 0.145 mg/ml, and a dissolution rate of 98.12% after 30 min. These findings demonstrated promising enhancement of MRT properties and increasing its oral bioavailability by 1.34-fold more than plain drug.

**Graphical Abstract:**

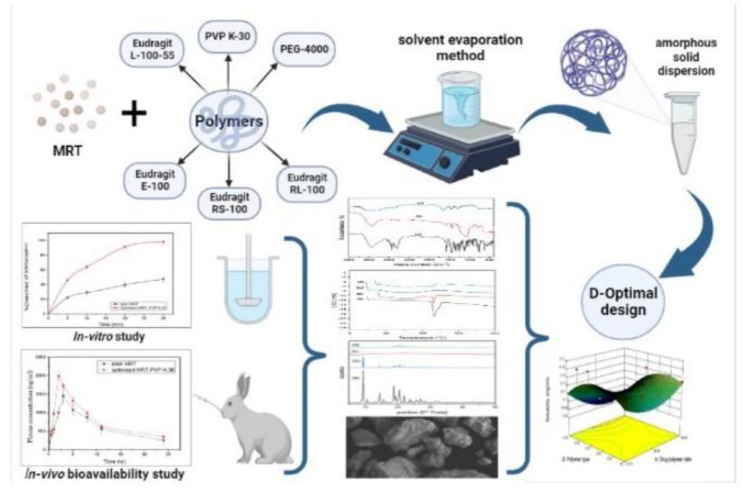

## Introduction

Depression is a widespread and hazardous health disorder that impairs a person’s ability to feel, think, and perform normally. It is anticipated that 3.8% of the population would suffer from depression, with 5% being adults and 5.7% of people above the age of 60 [1, 2]. Although imbalanced brain chemistry is one of the most frequently accepted hypotheses, depression is most probably caused by a complex interplay of the individual’s social, psychological, and biological circumstances [3]. Additionally, depression and general health are linked; for example, cardiovascular illness may contribute to depression and vice versa [1, 2]. Based on the severity and recurrence of the attacks in a time, healthcare professionals might consider visiting psychotherapist in parallel to antidepressant drugs such as selective serotonin reuptake inhibitors (SSRIs), tricyclic antidepressants (TCAs), and tetracyclic antidepressants (TeCAs) [1, 2].

Mirtazapine (MRT) is an atypical antidepressant licensed by the FDA in 1996 for the treatment of moderate to severe depression. This drug exhibits sedative, antiemetic, anxiolytic, and appetite-stimulating properties. Therapists often give MRT to depressed patients with sleep disturbance and/or underweight patients [4]. It was recently considered that it was safer than earlier medications; therefore, it replaced most previous antidepressants in depression medical therapy [5]. Mirtazapine has a poor solubility in water with a partition coefficient value of 2.9 and a bioavailability of around 50%. It was anticipated that boosting its water solubility might improves its oral bioavailability [6].

Solid dispersion (SD) technique represents one of the most attractive techniques to augment the wettability and solubility of drugs with poor water solubility [7, 8]. It is defined as a solid-state mixture containing one or more active compounds in the molecular state present in an inert carrier [9]. SDs can be prepared by dispersing drug molecules into different types of polymers using different methods. In addition to increasing the drug solubility and dissolution rate, SDs can reduce the drug particle size possibly to the molecular level, increasing porosity and wettability and changing particles from a crystal form to an amorphous form [10].

The aim of this investigation was to determine the optimum conditions to incorporate the MRT into various polymer types utilizing the D-optimal design targeting an MRT-SD formula with optimum physicochemical properties and improved oral absorption.

## Materials and methods

### Materials

Mirtazapine HCl was supplied from Mash Premiere for Pharmaceutical Industries (New Cairo City, Egypt). Eudragit (RL-100, RS-100, E-100, and L-100–55) were purchased from Sigma-Aldrich Chemical Co. (St. Louis, USA). Polyvinylpyrrolidone (PVP K-30) was obtained from BDH Chemicals Ltd. Poole, England. Polyethylene glycol (PEG 4000) was purchased from Fluka, Switzerland. Absolute ethanol was purchased from El-Gomhouria Co. For Trading Drugs, Chemicals & Medical Supplies (Cairo, Egypt).

### Methods

#### Experimental design

D-optimal design with two different types of factors (one factor was numeric at three levels (low, medium, and high), while the other factor was categoric with six levels) was established to estimate the influence of the independent variables (factors) on the dependent variables (responses) [11]. The design suggested 18 experiments (Table 2) to be conducted by using Design-Expert^®^ software. Table 1 shows the studied independent variables (drug/polymer percentage (A), polymer type (B)) and the dependent variables (loading efficiency (LE)% (*Y*_1_), aqueous solubility (*Y*_2_), and dissolution after 30 min (*Y*_3_)). The objective of the optimization stage was to obtain maximal solubility and dissolution after 30 min, with a LE% of 100%.

**Table 1:** D-optimal design values used in the optimization of the variables

	Response surface design					
Factors	Low (− 1)		Central (0)		High (+ 1)	
A: drug/polymer %	33.33		49.99		66.66	
B: polymer type	(1)	(2)	(3)	(4)	(5)	(6)
	Eudragit RL-100	Eudragit RS-100	Eudragit E-100	Eudragit L-100–55	PVP K-30	PEG 4000
Responses	Goal					
Y1: LE%	Target to 100%					
Y2: Solubility	Maximize					
Y3: Dissolution after 30 min	Maximize					

*LE%* loading efficiency %

##### Preparation of MRT solid dispersion

The MRT was loaded into Eudragit (RL-100, RS-100, E-100, and L-100–55), PVP K-30, and PEG 4000 with a drug/polymer percentage of 33.33%, 49.99%, and 66.66%, respectively. The loading process was achieved by the solvent evaporation method. The polymer was dissolved in ethanol using a magnetic stirrer at 25 °C for 2 h, and MRT was dispersed in the polymer solution to be dissolved. After obtaining a clear solution, the solvent evaporated at 60 °C using a magnetic stirrer. Finally, it was dried in a vacuum oven at 45 °C for 12 h to remove any solvent residuals to an acceptable limit level specified in the guidelines of the International Conference on Harmonization (ICH) Q3 (R5). Then, it was ground and sieved using a set of sieves to control the particle size [12].

#### Determination of LE%

The MRT loading efficiency was determined by producing a 1 mg/ml solid dispersion solution in ethanol. A sample was then centrifuged at 10,000 rpm for 10 min by utilizing a centrifuge (3–30 KS, Osterode, Germany). The specimen was diluted adequately and measured at max 293 nm using a UV–Vis spectrophotometer (Shimadzu, Japan). LE% was calculated by the following equation [13]:

1 $$\mathrm{LE}\%=\frac{\text{Practical drug content }}{\text{Theoretical drug content }}\times 100$$

#### Determination of aqueous solubility

An excess amount of a sample was mixed with 5 ml distilled water in sealed vials. The vials were then shaken in the water bath shaker for 48 h at 37 ± 1 °C. The resulting solution was passed through a 0.45 µm filter (Millipore). The MRT’s absorbance was measured using a UV–Vis spectrophotometer at *λ*_max_ 293 nm [6].

#### Determination of drug dissolution

The prepared formulas with a weight equivalent to 30 mg MRT were added to 500 ml of distilled water (pH 5.5) at 37 ± 1 °C, and a paddle of a USP dissolution apparatus II (DIS 6000, Switzerland) was turned at a speed of 50 rpm. The sink condition was maintained during the experiments. Samples of 2 ml were taken at specified sampling times of 5 min, 10 min, 20 min, and 30 min, then diluted and filtered through 0.45 µm Millipore filters before being analyzed using the UV–Vis spectrophotometer at *λ*_max_ 293 nm. Two milliliters of aliquot samples were collected and replaced to ensure that the sink condition was maintained during the dissolution process. Each experiment was reviewed three times [14].

#### Characterization of the optimum formula

##### Fourier-transform infrared spectroscopy

The Fourier transform infrared spectroscopy (FT-IR) analysis was conducted using a FT-IR spectrophotometer (IRAffinity-1; Shimadzu, Japan) to investigate the existence of distinctive MRT peaks, MRT peak shifting and masking caused by loading into PVP K-30, and the formation of new peaks. The samples were mixed with KBr, compacted onto a disc, then measured at a range of 4000 to 400 cm^−1^ with a 4 cm^−1^ resolution [15].

##### Differential scanning calorimetry

The differential scanning calorimetry (DSC) analysis was conducted using a differential scanning calorimeter (SDT Q600, USA). In a nitrogen environment, 2–4 mg samples were held to metal pans and warmed from 20 to 250 °C at a rate of 10 °C/min [12].

##### X-ray powder diffraction

The X-ray powder diffraction (XRPD) analysis was conducted by using the X-ray diffractometer (XDS 2000, USA). The samples were placed in the holder before being exposed to monochromatized CuK radiation at 30 kV and 30 mA. The step size within an angle of 2*θ* is over a range of 5–50° [16].

##### Scanning electron microscopy

Scanning electron microscopy (SEM) was performed to evaluate the morphology of tested samples by using the scanning electron microscope (JSM-6360, Japan). Prior to inspection, the samples were coated with gold. A double-sided sticky strip was used to hold about 1 mg of each sample to a sample holder. SEM pictures were captured using an accelerating voltage of 15 V [17].

#### In vivo bioavailability study of the optimum formula

##### HPLC analysis of rabbits’ plasma samples

A stock solution of the drug was prepared by using a methanolic solution of MRT at a concentration of 1 mg/ml. Serial dilutions were spiked into blank plasma to obtain concentrations of 200 ng/ml, 400 ng/ml, 600 ng/ml, 800 ng/ml, and 1000 ng/ml. Analysis of the samples was performed using the HPLC system (Waters Alliance 2690, USA). Phosphate buffer (pH 3.9) and acetonitrile (90:10) were used as a mobile phase, and rabbits’ plasma samples were injected by the isocratic elution into the XTerra C18 column (4.6 mm × 100 mm, 5 µm). The flow rate was 1 ml/min at ambient temperature. The wavelength of the drug (*λ*_max_ 293 nm) was detected by the photodiode array detector (Waters 996 HPLC Photodiode Array Detector, USA) [18].

##### Animal study

The study was conducted in the animal unit at Zagazig University in Egypt. Ten male white rabbits weighing between 1600 and 1800 g were kept in a room with a 12-h light and dark cycle. They went without food for 24 h prior to the study and remained hungry for 6 h after the drug was given, but drinking water was allowed [19]. The study was conducted in agreement with the Guide for the Care and Use of Laboratory Animals [20] and the Zagazig University, Faculty of Pharmacy, Institutional Animal Care and Use Committee (IACUC) guidelines (Approval number: ZU-IACUC/3/F/105/2021).

##### Animal handling and drug administration

Rabbits were separated into two groups in a single-blind, randomized investigation. Group I got plain MRT, while group II received the optimum formula. A dose was 15 mg/kg, which was given by mouth through a pharyngostomy tube (4 French) [21]. At 0 h, 0.5 h, 1 h, 2 h, 3 h, 5 h, 8 h, 11 h, and 24 h, 0.5 ml samples were collected from the rabbit orbital vein in heparinized tubes. The plasma was separated by centrifugation at 4000 rpm for 10 min and frozen at − 20 °C till needed. MRT concentrations were assessed by using the HPLC at *λ*_max_ of 293 nm [21, 22].

##### Determination of pharmacokinetic parameters

The PKSolver^®^ software was used to compute the primary parameters such as maximum plasma concentration (*C*_max_), time to achieve this concentration (*T*_max_), area under the plasma concentration–time curve (AUC_0–*t*_), area under the curve from time zero to infinity (AUC_0–∞_), and the half-life time (*t*_1/2_). Oral bioavailability of the optimum formula was calculated relative to the plain drug, according to the subsequent equation [21]:

2 $$\begin{aligned}\mathrm{Relative}\; \mathrm{bioavailability}\; \left(\%\right)=\big({\mathrm{AUC}}_{0-t} \left(\mathrm{optimum}\; \mathrm{formula}\right)\\/{\mathrm{AUC}}_{0-t} \left(\mathrm{plain}\; \mathrm{drug}\right)\big)\times 100\end{aligned}$$

## Results and discussion

### Analysis of D-optimal design

D-optimal design experiments were implemented to assess the independent variables that might have an impact on the dependent responses. The design results showed that all responses were intimately correlated to the selected factors (Table 2). This was indicated by the significant *P* values obtained through the analysis of variance (ANOVA) test (Table 3) [23].

**Table 2:** Experiments suggested by the D-optimal design

Formula	Drug/polymer (%)	Polymer type	Loading efficiency (%)	Solubility (mg/ml)	Dissolution after 30 min (%)
1	33.33	PVP K-30	104.93 ± 2.82	0.175	98.12
2	49.99	Eudragit L-100–55	101.12 ± 3.10	0.123	69.69
3	33.33	PEG 4000	101.37 ± 3.10	0.097	88.98
4	33.33	Eudragit L-100–55	103.32 ± 0.77	0.189	78.68
5	66.66	Eudragit RS-100	105.44 ± 3.10	0.128	35.23
6	66.66	Eudragit E-100	104.83 ± 1.59	0.122	27.47
7	66.66	Eudragit L-100–55	103.32 ± 2.55	0.128	45.23
8	49.99	Eudragit RS-100	102.75 ± 1.72	0.114	39.38
9	66.66	PEG 4000	104.53 ± 4.68	0.099	37.46
10	49.99	Eudragit RS-100	102.75 ± 3.56	0.114	39.38
11	66.66	Eudragit RL-100	105.84 ± 3.02	0.125	37.14
12	66.66	PVP K-30	93.00 ± 2.82	0.094	32.12
13	33.33	Eudragit RS-100	102.65 ± 2.38	0.117	50.09
14	49.99	PEG 4000	100.73 ± 3.10	0.093	59.41
15	33.33	Eudragit E-100	84.23 ± 3.10	0.112	78.9
16	33.33	Eudragit RL-100	102.59 ± 1.19	0.121	62.14
17	49.99	PVP K-30	102.34 ± 2.66	0.102	57.59
18	49.99	Eudragit L-100–55	101.12 ± 3.12	0.123	69.69

**Table 3:** ANOVA results for the dependent variables

	Model	*R*^2^	Adjusted *R*^2^	Predicted *R*^2^	Adequate precision	*P* value	*F* ratio	
*Y*_1_^a^	Linear	0.37	0.28	0.11	5.94	0.0306	4.44	Significant
*Y*_2_^b^	Quadratic	0.65	0.51	0.70	6.9	0.0147	4.55	Significant
*Y*_3_^c^	2FI	0.84	0.81	0.75	14.74	0.0001	25.92	Significant

^a^Loading efficiency %

^b^Aqueous solubility

^c^Dissolution after 30 min

#### Influence of the variables on LE% (*Y*_1_)

The percentage of drug loaded into the polymer represents one of the most significant factors to determine the drug incorporation efficacy into the polymer in the SD formulas. When the drug and polymer are in intimate molecular contact, the drug molecules are inserted into the spaces between the loosening polymeric chains. The solvent evaporation method is successful for MRT loading using ethanol as an organic solvent. This is owing to the great solubility of the drug in the ethanol which successfully converts the drug into the molecular state and then makes inter- and intra-weak bonds between polymer chain molecules and drug molecules [24]. The LE% was affected by changing the polymer used for drug loading by changing the drug-to-polymer percentage. The MRT was efficiently loaded into Eudragit RL-100, RS-100, E-100, L-100–55, PEG 4000, and PVP K-30 by using different drug/carrier ratios. It was found that the drug loading percentage for all MRT-SD formulas was in an acceptable range, as it varied from 83.2% (F12) to 105.84% (F11) (Table 2) [21].

**Fig. 1:**
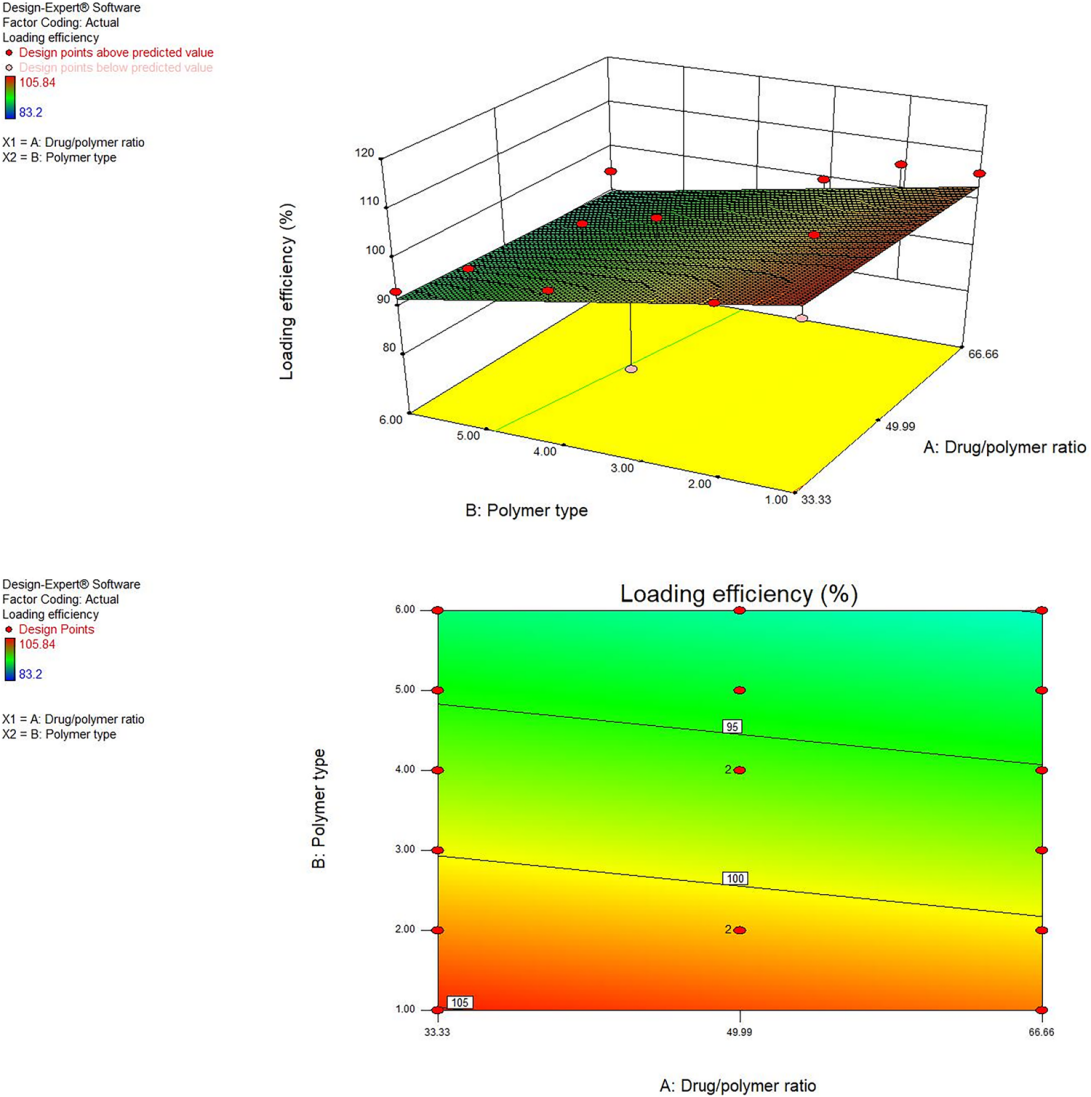
Response 3D plot and contour plot for the influence of drug/polymer ratio in percentage (A) and polymer type (B) on the LE% (*Y*_1_)

In the present work, it was noticed that the drug/polymer percentage (A) did not significantly impact the LE% of MRT (*P* > 0.05) while the polymer type (B) has a significant influence on the drug’s LE% (*P* < 0.05). The LE% was improved after loading the MRT into different polymers by the following subsequent order: PEG 4000 < PVP K-30 < Eudragit L-100–55 < Eudragit E-100 < Eudragit RS-100 < Eudragit RL-100. The MRT loaded into Eudragit RL-100, RS-100, and E-100 showed the highest LE%, which increased by increasing the drug ratio to 66.66%. This might occur as a result of the high electrostatic bonds that exist between these anionic polymers and the positively charged MRT molecule [25]. The 3D response surface plot and contour plot were used to analyze the influence of the independent factors on LE% (Fig. 1).

In conclusion, results of studying the influence of the two independent factors on the LE% (*Y*_1_) showed that loading of MRT into Eudragit RL-100 at a percentage of 66.66% by solvent evaporation method gave the maximum LE% of 105.84%.

#### Influence of the variables on aqueous solubility (*Y*_2_)

Saturation aqueous solubility studies were done on the pure MRT and the solid dispersion of MRT with Eudragit RL100, RS100, E100, L100-55, PVP K-30, and PEG 4000 at the percentage of 49.99%, 66.66%, and 33.33%. The results of the saturated aqueous solubility research show that plain MRT has low water solubility (0.092 mg/ml), whereas prepared samples have higher drug solubility (Table 2). This could be attributed to drug’s particle wetting and localized solubilization by the hydrophilic polymers [26].

The type of polymer used showed a significant effect on MRT solubility (*P* < 0.05). The MRT solubility improved when loaded into different polymers by the subsequent order: PEG 4000 < Eudragit RL-100 < Eudragit RS-100 < Eudragit E-100 < Eudragit L-100–55 < PVP K-30. Among various MRT solid dispersions, formulas prepared using PVP K-30 showed higher aqueous solubility, while others showed minimal solubility improvement. Such enhancement of solubility might be attributed to the improved wettability of MRT induced by the formation of intermolecular hydrogen bonds between MRT and PVP K-30 polymer [27]. Additionally, the increased water solubility of MRT in solid dispersions might be explained by the lower surface tension effect of the PVP K-30 which improved drug wetting in the dissolution medium [28].

**Fig. 2:**
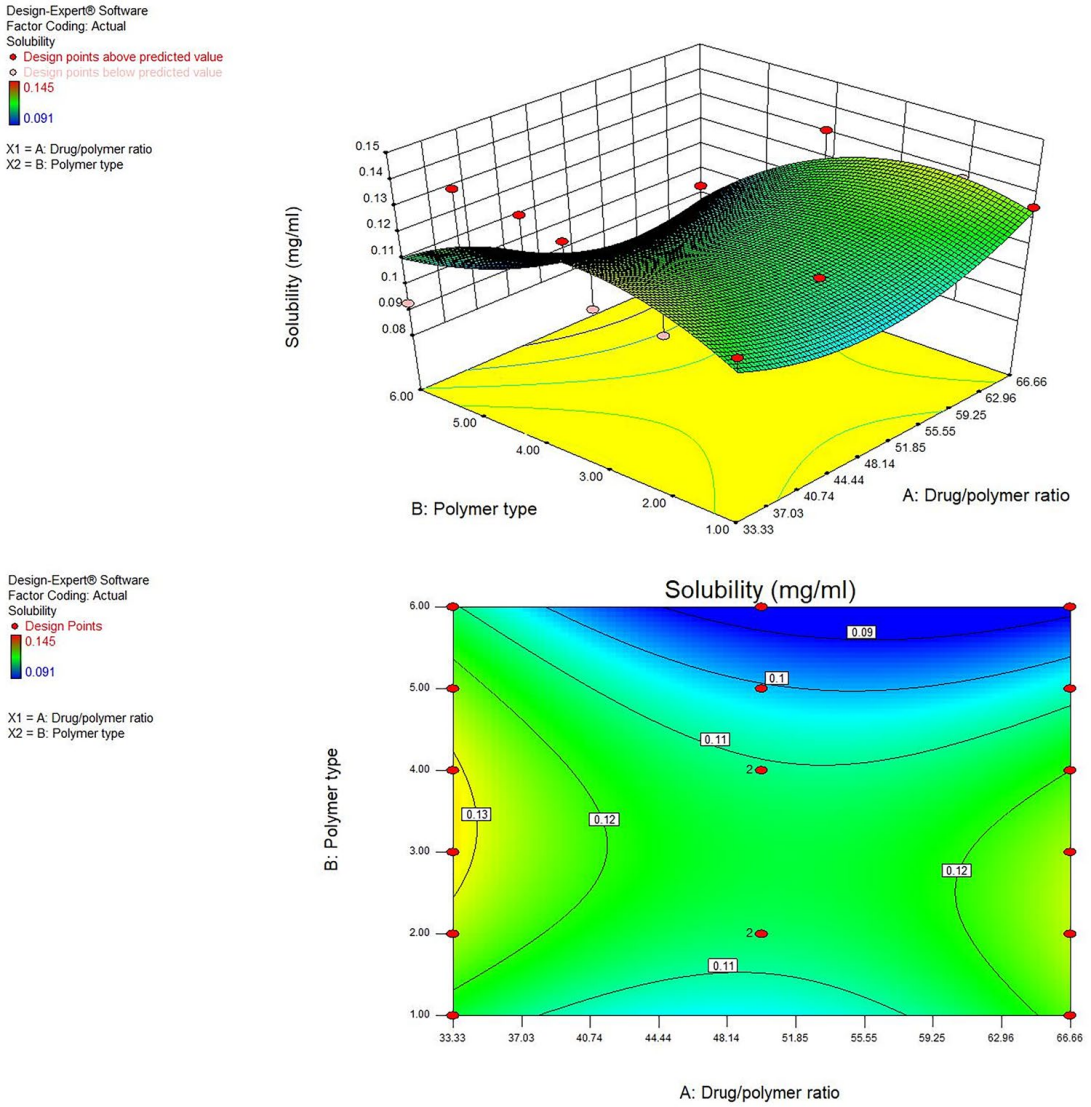
Response 3D plot and contour plot for the influence of drug/polymer ratio in percentage (A) and polymer type (B) on the aqueous solubility (*Y*_2_)

The drug-to-polymer ratio also has a significant influence on the drug aqueous solubility (*P* < 0.05). The 3D response surface plot and contour plot were used to analyze the influence of the independent factors on solubility (Fig. 2). Increasing solubility of MRT was best achieved through increasing the polymer ratio since the highest solubility was achieved as a result of loading MRT at a percentage of 33.33% (Fig. 2). This could be related to the high concentration of hydrophilic polymers [29].

In conclusion, the influence of variables on MRT aqueous solubility was studied, and it was found that loading MRT into PVP K-30 in the percentage of 33.33% using solvent evaporation method enhanced MRT saturation solubility from 0.092 mg/ml to roughly 0.145 mg/ml.

#### Influence of the variables on drug dissolution after 30 min (*Y*_3_)

**Fig. 3:**
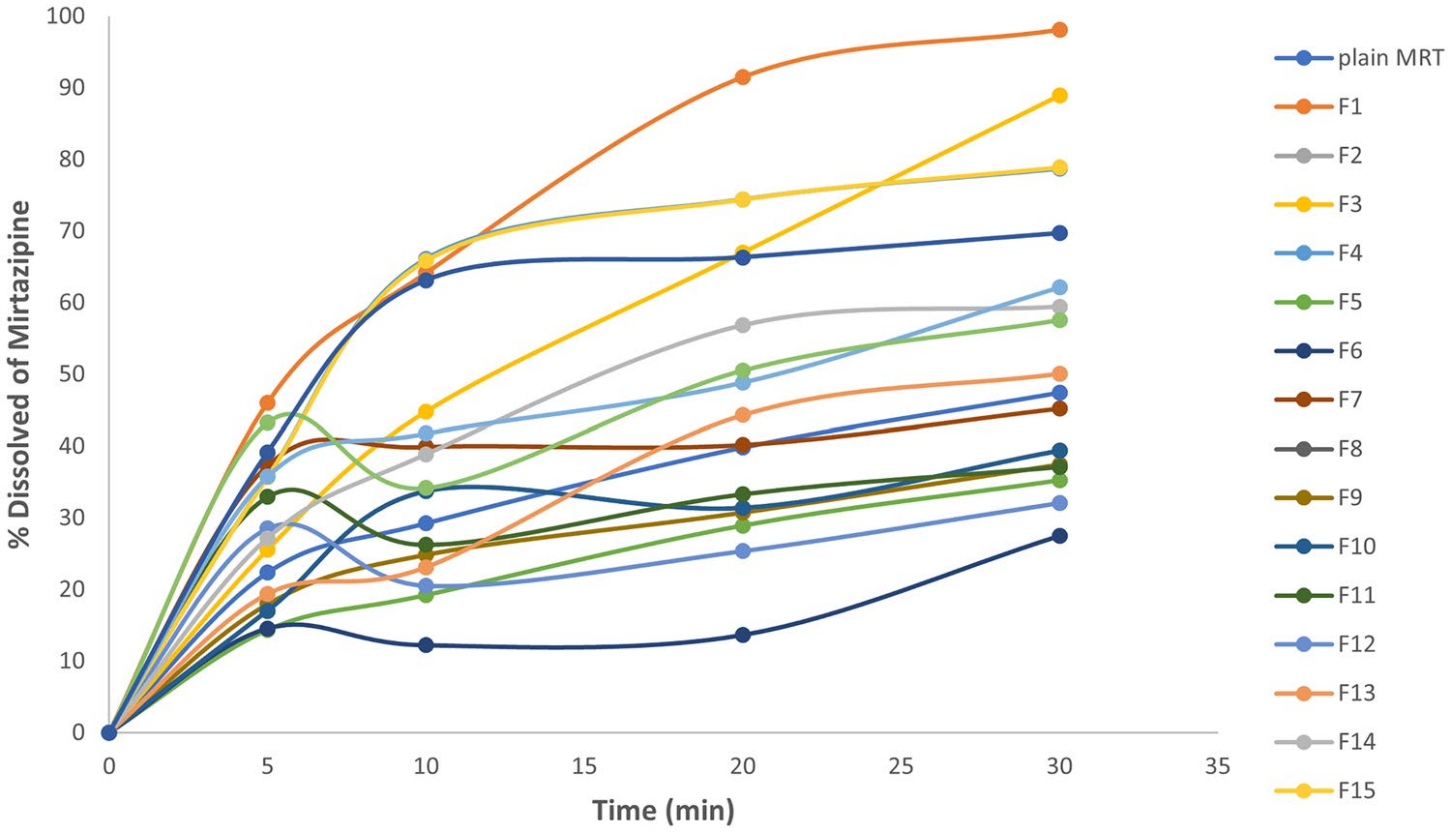
The dissolution pattern of plain MRT and MRT loaded into different polymers in the formulas selected by D-optimal design

As shown in Table 2 and Fig. 3, the percentage of MRT dissolute from the prepared formulas was compared to that of plain MRT. The boosted dissolution rate of MRT by solid dispersion is mostly due to the higher degree of hydrophilicity caused by the incorporation of the drug particles into the hydrophilic polymers. This augmented the drug wettability and subsequently improved its solubility, accordingly saving the need of energy to break down the crystal lattice of a drug in the dissolution process [30].

Furthermore, the enhanced dissolution of MRT-SD is owing to the drug’s amorphous form, contrasting to the crystalline state of the plain drug [26]. The amorphous drug systems are thermodynamically unstable, having higher energy and greater molecular motion, which lead to a great apparent solubility and dissolution rate [31].

The type of polymer used in the preparation of the MRT solid dispersion showed a significant impact on the drug dissolution rate (*P* < 0.05). The dissolution rate of MRT has been enhanced by loading the drug into the hydrophilic polymers in the following ascending order: RS-100 < RL-100 < E-100 < L-100–55 < PEG 4000 < PVP K-30. The highest improvement in MRT dissolution rate occurred when MRT was loaded into PVP K-30, and this was influenced by the presence of polar function groups such as N–H and C = O groups on the pyrrolidine moiety of the PVP K-30 which readily form hydrogen bonds with the water molecules. The strength of the bonds formed between the water and polymer or drug molecules may be similar to or greater than that formed between the molecules of the solid dispersions. Following contact with water, the polymer and the amorphous drug molecules were dissolved and the hydrogen bonds between the drug and PVP complex were disrupted [32]. In addition, the high dissolution rate of MRT-PVP K-30 dispersions may be attributable to the great ability of solubilization and wetting of the PVP K-30 compared to the other polymers [33]. In conclusion, the improvement of the dissolution of drugs from MRT-PVP K-30 is based mainly on three different mechanisms: the wettability of the drug (which is improved by direct contact with the PVP K-30), the reduction in the particle size and increased surface area, and the conversion of the crystalline state to the more soluble amorphous state. Also, the PVP K-30 has been extensively studied as an efficient carrier in solid dispersions of various drugs due to its ability to retard and inhibit recrystallization of drugs due to its high glass transition temperature [28].

**Fig. 4:**
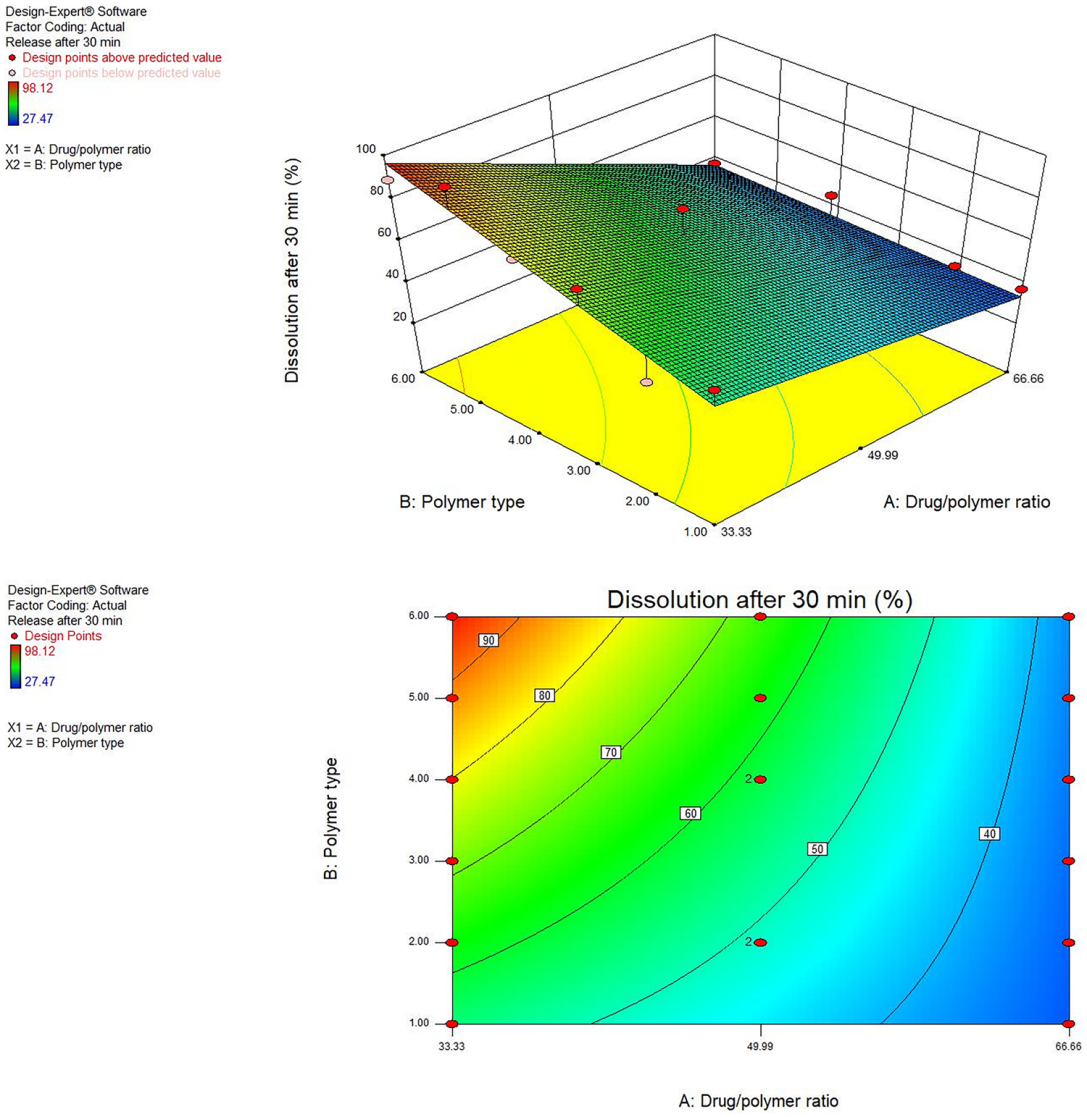
Response 3D plot and contour plot for the influence of drug/polymer ratio in percentage (A) and polymer type (B) on the dissolution after 30 min (*Y*_3_)

The drug-to-polymer percentage has also a significant impact on the drug dissolution rate (*P* < 0.05). The dissolution rate was increased with the increase of the PVP K-30 percentage in the preparation [34]. The MRT dissolution rate from solid dispersion preparation showed the highest dissolution rate when it was loaded by 33.33% of drug/polymer. The 3D response surface plot and contour plot were used to analyze the influence of the factors on the LE% (Fig. 4).

It was concluded that the MRT loaded into PVP K-30 at the percentage of 33.33% by solvent evaporation method increased the dissolution rate of MRT after 30 min from 47.4 to 98.12%.

**Fig. 5:**
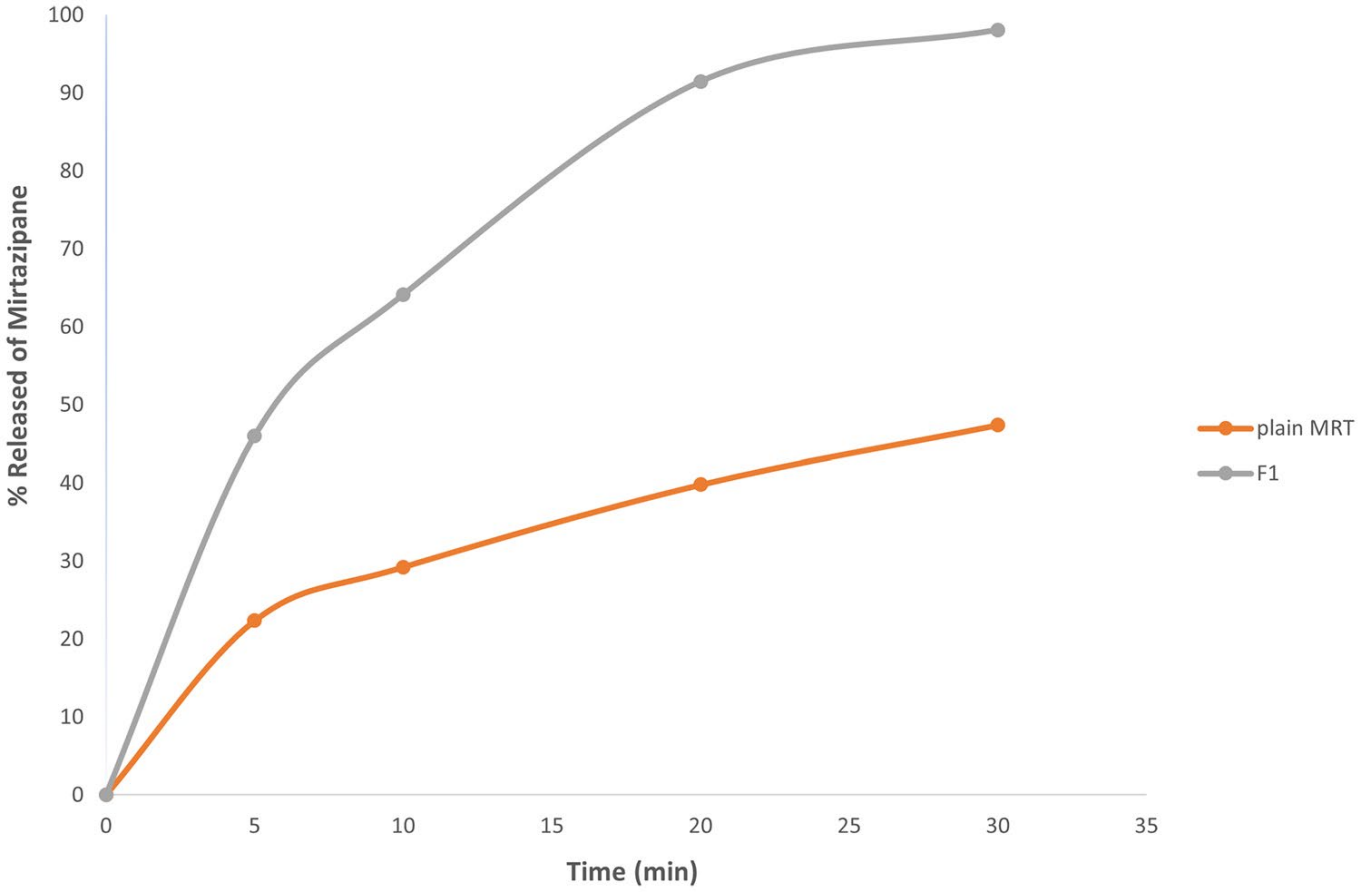
The dissolution pattern of the plain MRT and the optimum MRT-PVP K-30

The dissolution of MRT-PVP K-30, which has an amorphous structure, was much faster than the crystalline plain drug. The amount of plain MRT dissolution after 30 min was 47%, whereas the maximum amount of MRT-PVP K-30 dissolution after 30 min was 98.12% (Fig. 5).

It was concluded that the MRT loaded into PVP K-30 at the percentage of 33.33% by solvent evaporation method increased the dissolution rate of MRT after 30 min from 47.4 to 98.12%.

### Validation of the optimization process

The optimization process was done to find the drug/polymer percentage (A) and the type of polymer (B) values which maximize aqueous solubility (*Y*_2_) and dissolution after 30 min (*Y*_3_), while targeting LE% (*Y*_1_) to reach 100% (Table 2). The optimum formula selected by the design showed a LE% of 100.93%, solubility of 0.175 mg/ml, and dissolution rate of 98.12%. To confirm the optimization process, three samples were prepared according to the predicted level obtained by the D-optimal design. The consistency between the expected and the actual findings indicates the validity of design goals (Table 4) [21].

**Table 4:** Predicted and observed values of the optimum formula of MRT-PVP K-30

Factors	Optimum level		
A: drug/polymer (%)	33.33		
B: polymer type	PVP K-30		
Responses	Expected	Observed	Residual^a^
*Y*_1_: LE%	94.56	100.93	− 6.37
*Y*_2_: aqueous solubility (mg/ml)	0.124	0.175	− 0.021
*Y*_3_: dissolution after 30 min (%)	88.15	98.12	− 9.97

*LE%* loading efficiency %

^a^Expected–observed

### Characterization of the optimum formula

#### FT-IR

**Fig. 6:**
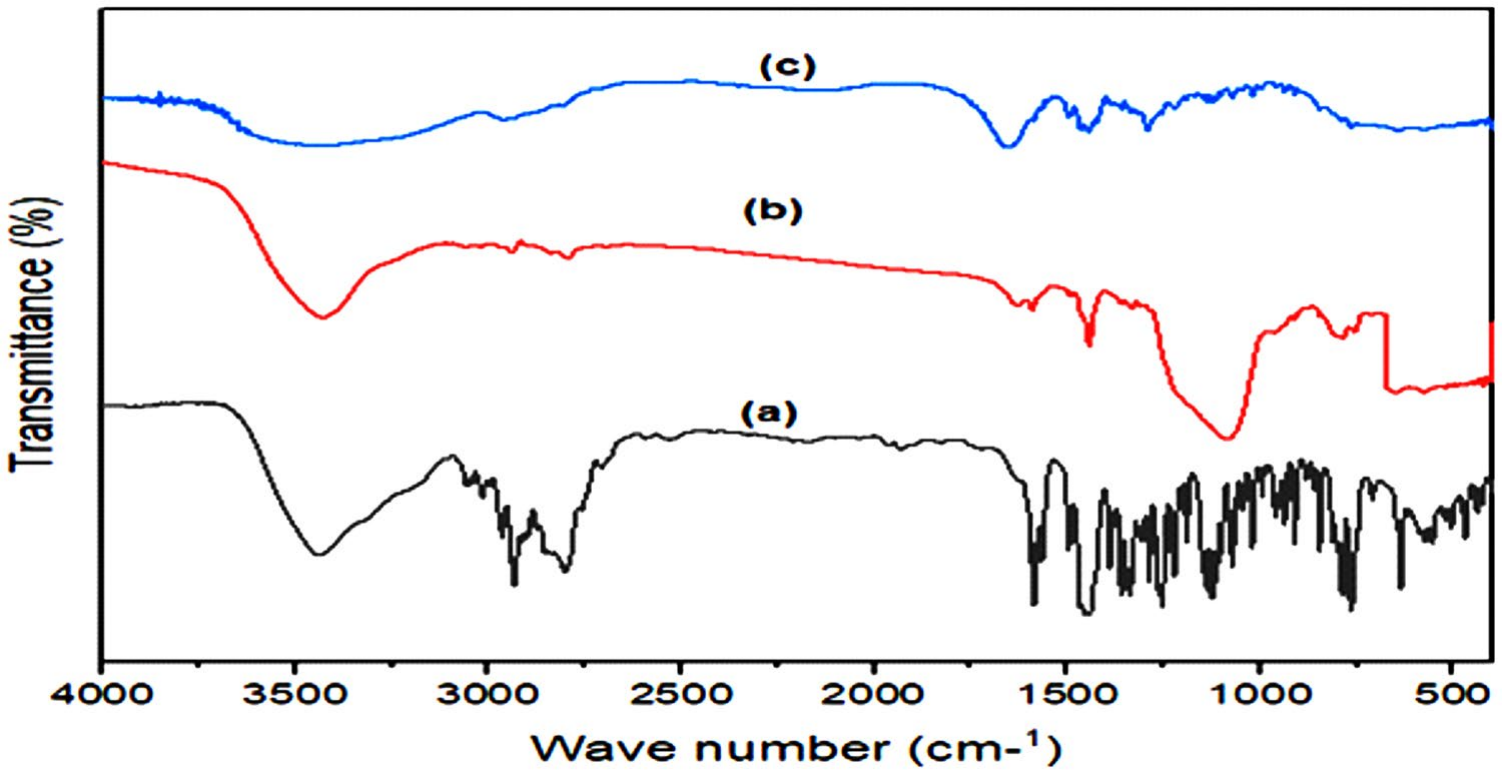
FT-IR analysis of **a** plain MRT, **b** plain PVP K-30, and **c** optimum MRT-PVP K-30

FT-IR of plain MRT exhibited great peaks at 3439 cm^−1^, indicating N–H stretching, a band at 2931 cm^−1^ arising from a methyl group connected to N_2_ atom, and bands for C–C stretching of the phenyl group at 1587 cm^−1^ and 1450 cm^−1^. The bands at 1336–1253 cm^−1^ were produced by primary aromatic amines with N directly linked to the ring. The benzene ring C–H arises in the 1359–1074 cm^−1^ range (Fig. 6a) [35].

In Fig. 6b, the spectrum of PVP K-30 reveals a major distinctive peak around 1138 cm^−1^, denoting a C–N stretching vibration and a band at 1490 cm^−1^ attributed to C = O. An important broad was also visible at 3435 cm^−1^ that was due to O–H stretching vibrations of absorbed water [36, 37].

Comparison of the FT-IR spectra of plain MRT and the optimized MRT-PVP K-30 shows that FT-IR spectra of the optimum formula exhibit band shifts and broadening; this is owing to the pyrrolidone component of PVP K-30, which contains two groups (= N– and C = O) that might possibly develop hydrogen bonds with the MRT. However, steric hindrance prevents the nitrogen atom from participating in intermolecular interactions, making the carbonyl group more attractive for hydrogen bonding. A combined effect of interactions and reduced mobility leads to production of stable amorphous structure of MRT inside the polymer during the solid dispersion preparation (Fig. 6c) [37, 38].

#### DSC

**Fig. 7:**
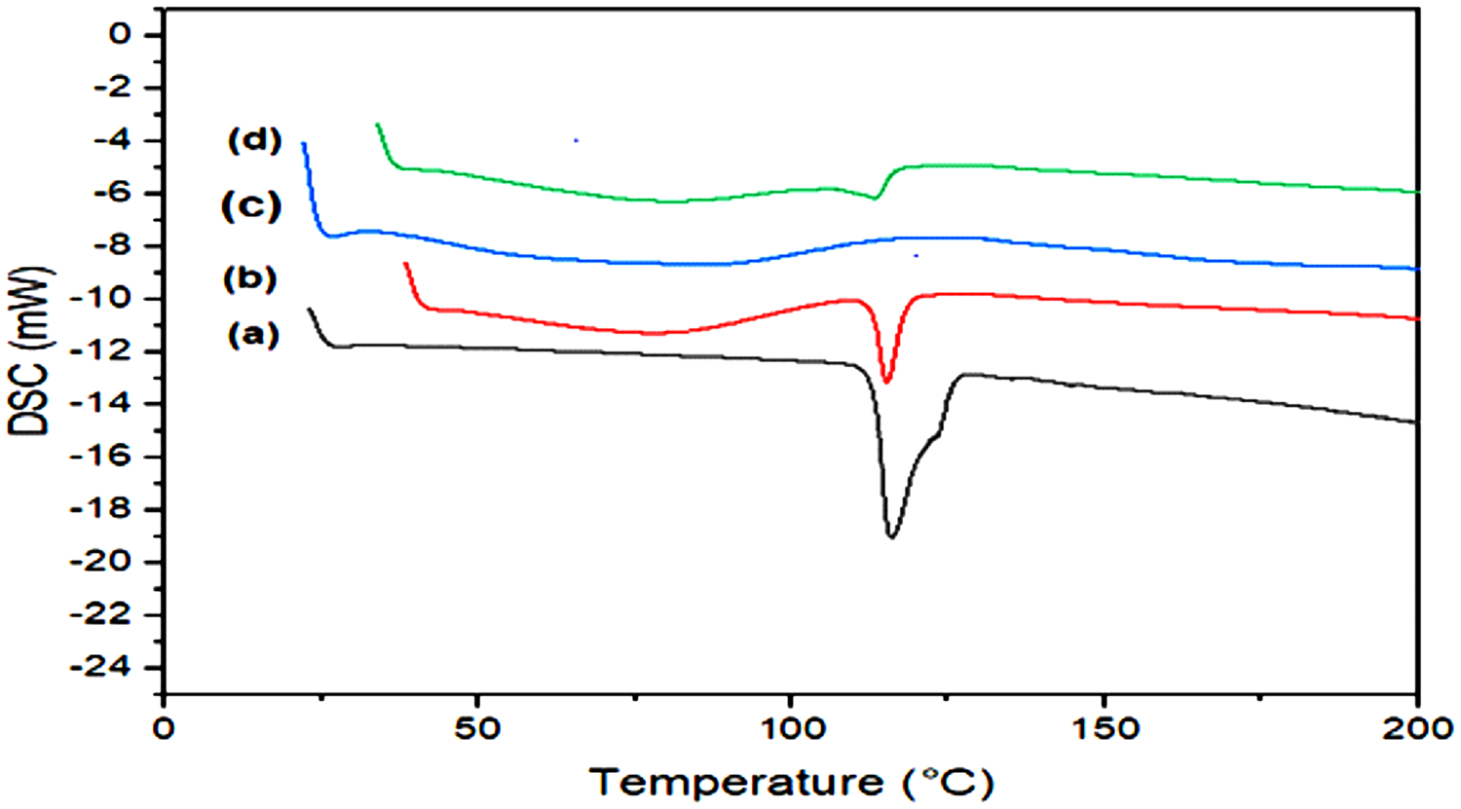
DSC analysis of **a** plain MRT, **b** physical mixture of MRT/PVP K-30, **c** plain PVP K-30, and **d** optimum MRT-PVP K-30

The DSC spectrum of plain MRT’s exhibits a notable strong endothermic peak at 115 °C, demonstrating the crystalline structure of the drug (Fig. 7a). The curve of plain PVP K-30 demonstrates its amorphous nature (Fig. 7c). Moreover, in Fig. 7b, the peak of drug crystals can also be observed in the physical mixture. The optimum formula of MRT-PVP K-30 exhibited a considerable reduction in the MRT peak, revealing the replacing of the majority of the crystalline structure of the plain by the amorphous form. This may be due to the homogeneous dispersion of the MRT in the polymer crust (Fig. 7d) [36].

#### XRPD

**Fig. 8:**
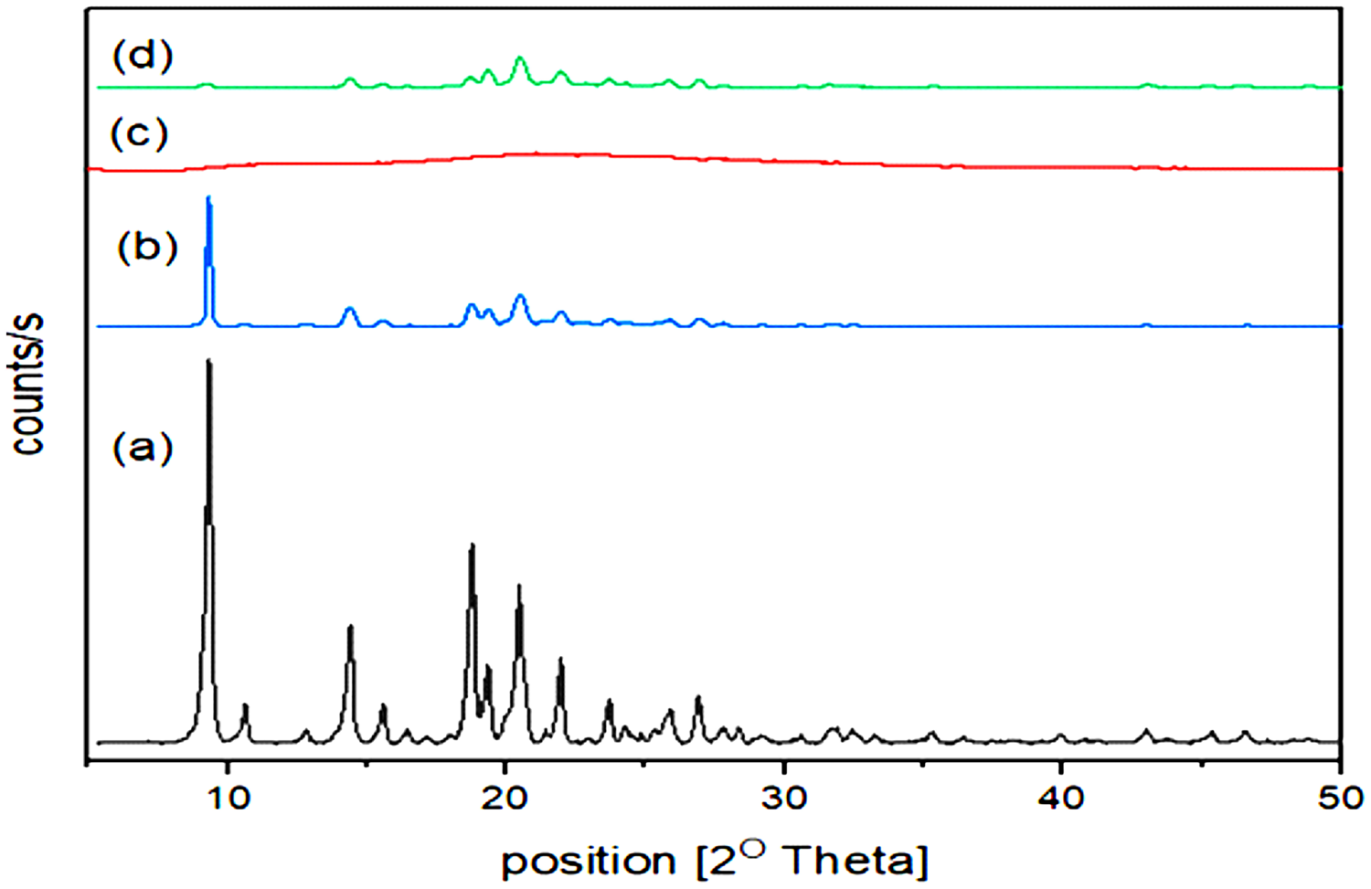
PXRD analysis of **a** plain MRT, **b** physical mixture of MRT/PVP K-30, **c** plain PVP K-30, and **d** optimum MRT-PVP K-30

The plain MRT’s XRPD showed prominent and strong diffraction peaks arising throughout the range of 2*θ* values of 9.5°, 14.5°, 19.0°, and 20.7°, indicating the MRT’s very crystalline nature (Fig. 8a). When MRT and PVP K-30 were physically mixed, the same peaks were observed (Fig. 8b). In contrast to prior results, amorphous PVP K-30 exhibited no diffraction peaks attributable to its lack of crystallinity (Fig. 8c). After the integration of MRT into PVP K-30, the XRPD pattern reveals a significant decrease in the distinctive MRT peaks in the SD systems, owing to the dispersion of a greater proportion of the drug in the solid state. Additionally, a significant decrease in distinctive peaks implies the drug amorphous form (Fig. 8d). The PXRD diffractograms showed clearly how the polymers changed the drug’s crystalline nature into a nearly completely amorphous one, enhancing the dissolution of the drug [39].

#### SEM

**Fig. 9:**
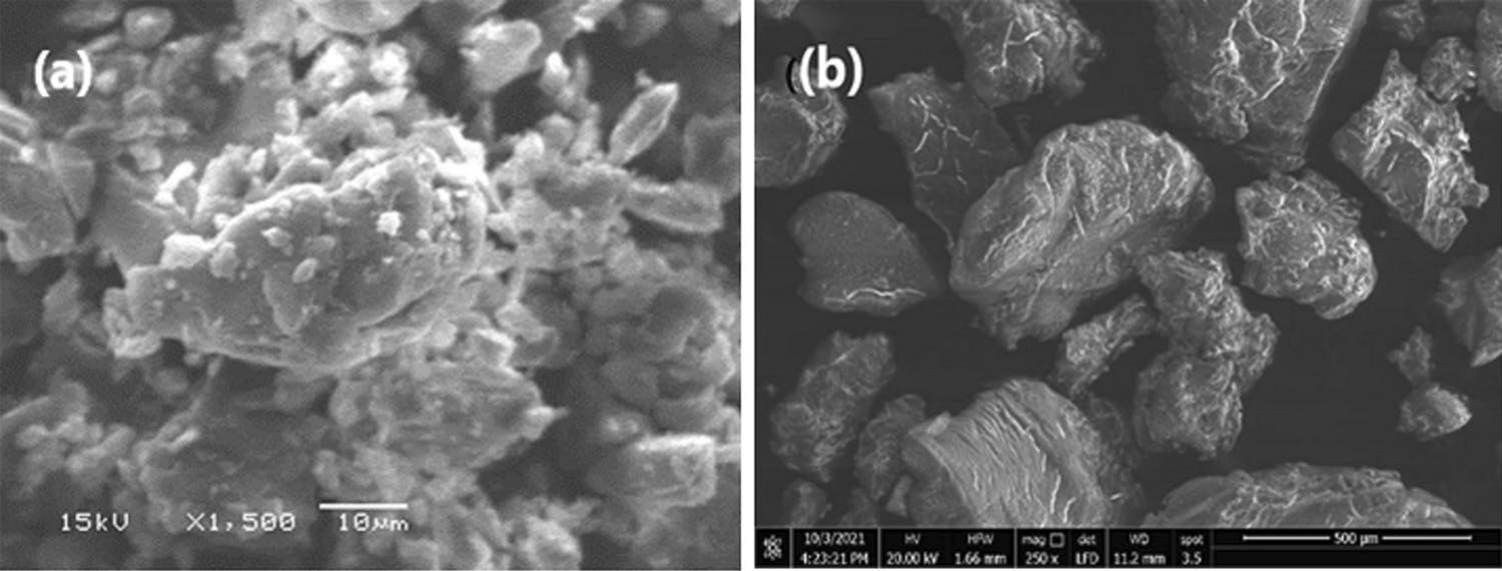
SEM of **a** plain MRT and **b** optimum MRT-PVP K-30

The SEM images examine the surface properties of plain MRT and the optimum MRT-PVP K-30 formula (Fig. 9). The image demonstrates the crystalline structure of plain MRT (Fig. 9a), which vanished when incorporated into the PVP K-30 (Fig. 9b), showing an amorphous form of the loaded MRT. This alteration was the main reason for the loaded MRT’s rapid dissolution.

### In vivo bioavailability study

**Fig. 10:**
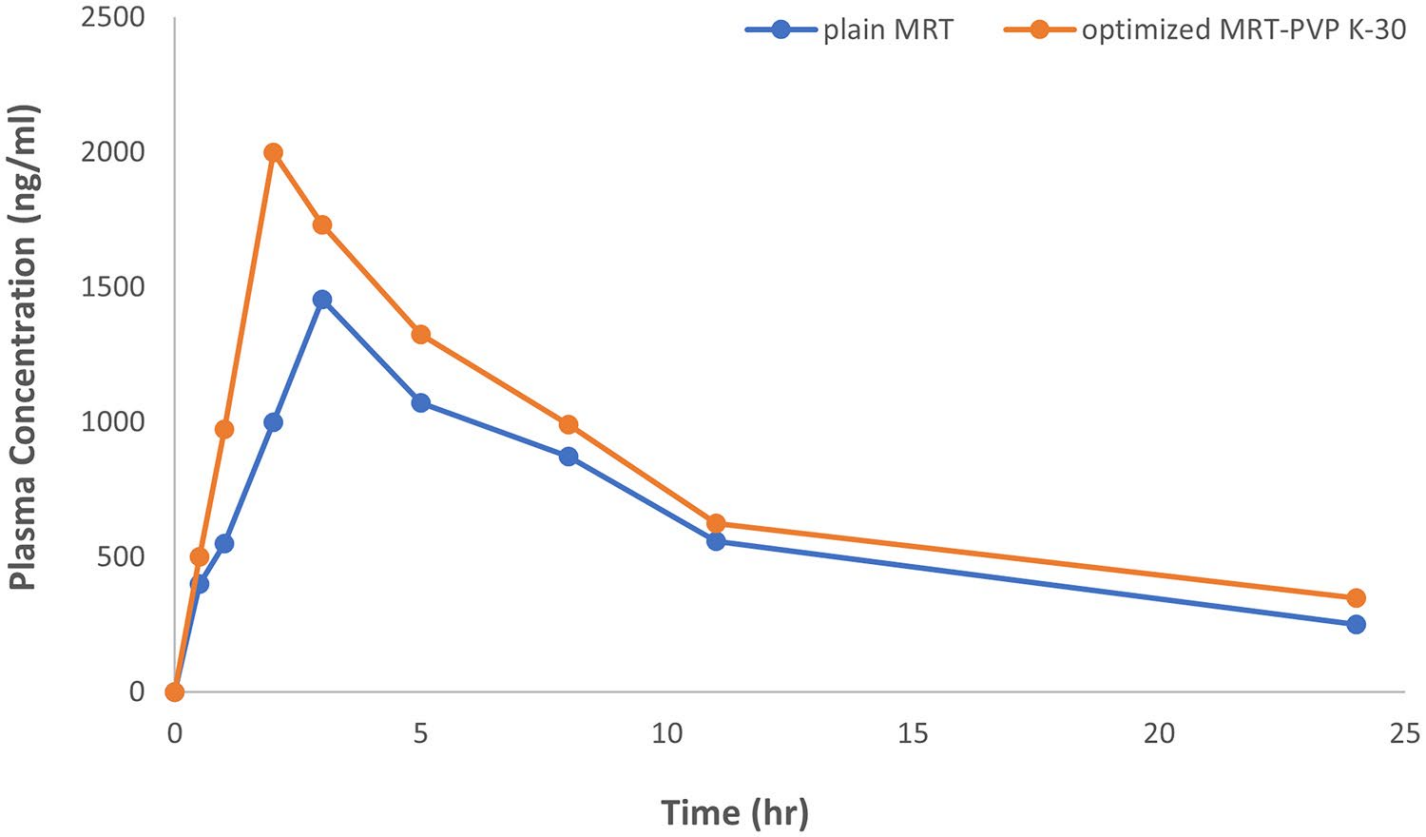
Plasma concentration–time curve of plain MRT and the optimum MRT-PVP K-30

The HPLC approach was conducted to quantitatively measure drug concentrations in rabbit’s plasma. The standard curve of MRT in rabbits’ plasma exhibited high linearity (*R*^2^ = 1) [40]. The plasma concentration–time curve demonstrates the increasing plasma level after loading MRT into the PVP K-30 (Fig. 10). The values of *C*_max_, *T*_max_, AUC_0–*t*_, AUC_0–∞_, and *t*_½_ of the optimum MRT-PVP K-30 show significant improvement compared to those of the plain MRT (*P* < 0.05). The statistical comparison was carried out in accordance with Student’s *t* test by using SPSS® software trial. The optimum formula of MRT-PVP K-30 boosted the drug oral absorption with a relative bioavailability of 134.5% (Table 5). Consequently, the optimum formula implies that a lower therapeutic dose may be utilized to have a similar clinical impact with lower adverse effects [41]. In conclusion, this study reveals the PVP K-30’s ability to overcome the MRT’s poor solubility, increasing the drug oral absorption.

**Table 5:** Pharmacokinetic parameters of plain MRT and optimum MRT-PVP K-30

Parameters	Plain MRT	Optimum MRT-PVP K-30
*C*_max_ (ng/ml)	1455.21 ± 167.10	2062 ± 122.37
*T*_max_ (h)	3 ± 0.00	2 ± 0.00
*t*_½_ (h)	8.95 ± 0.83	11.72 ± 0.92
AUC_0–*t*_ (ng/ml/h)	15,205.45 ± 1619.65	19,472.7 ± 599.55
AUC_0–∞_ (ng/ml/h)	18,450.16 ± 2128.24	24,878.13 ± 1047.01
Relative bioavailability (%)	–	134.8

*C*_max_ is the maximum plasma concentration, *T*_max_ is the time required to reach a maximum plasma concentration, *t*_1/2_ is the elimination half-life, AUC_0–*t*_ is the area under the plasma concentration–time curve, and AUC_0–∞_ is the area under the curve from time zero to infinity

## Conclusion

Utilizing the solvent evaporation technique, the drug under research was efficiently incorporated in SD formulations using Eudragit RL-100, RS-100, E-100, L-100–55, PVP K-30, and PEG 4000. The D-optimal design was chosen to assess the impact of the independent variables on the dependent responses. The amorphous form of MRT-PVP K-30 was confirmed by physiochemical analysis. Findings showed that the optimum formula was achieved by preparing the MRT solid dispersion with PVP K-30 at a drug/polymer percentage of 33.33% using the solvent evaporation method, which massively increased the drug’s oral bioavailability by reducing its extensive low water solubility. The bioavailability study on rabbits’ plasma sample confirmed these results by showing a 1.34-fold increase in the drug oral absorption from the optimum MRT-PVP K-30 formula more than that of the plain MRT, with a relative bioavailability of 134.5%.

## Data Availability

The datasets generated during and/or analyzed during the current study are available from the corresponding author on reasonable request.
